# MicroRNAs and vascular damage in chronic kidney disease: advances and clinical implications

**DOI:** 10.1590/2175-8239-JBN-2024-0223en

**Published:** 2025-05-19

**Authors:** Regiane Stafim da Cunha, Carolina Amaral Bueno Azevedo, Guilherme Miniskiskosky, Paulo Cézar Gregório, Andréa Emilia Marques Stinghen

**Affiliations:** 1 Universidade Federal do Paraná, Laboratório de Nefrologia Experimental, Departamento de Patologia Básica, Curitiba, PR, Brazil.

**Keywords:** Renal Insufficiency, Chronic, Endothelial Dysfunction, Vascular Calcification, MicroRNAs

## Abstract

Chronic kidney disease (CKD) is closely associated with endothelial dysfunction and vascular calcification, which are major contributors to the development of cardiovascular disease in this population. MicroRNAs (miRNAs) are a group of non-coding RNAs that regulate gene expression and other cellular processes. Recent studies have demonstrated that changes in the levels of several miRNAs are associated with the progression of renal dysfunction. Patients with CKD have reduced levels of miR-126, a microRNA produced by the endothelium that has an atheroprotective function. Reduced miRNA levels that inhibit vascular calcification, such as miR-133a and miR-204-5p, are also found in patients with CKD. These changes may contribute to vascular dysfunction in these patients. Therefore, understanding the profile of microRNAs in the context of CKD may be important for the identification of new biomarkers and potential therapeutic targets. Given the growing relevance of microRNA analysis, this review addresses recent advances in the study of microRNAs related to vascular dysfunction in CKD and their potential applications in translational clinical practice.

## Introduction

Chronic kidney disease (CKD) is characterized by gradual and irreversible loss of kidney function and is highly associated with hypertension and diabetes. Complications and cardiovascular disease (CVD) are intimately associated with CKD and the main risk factors for mortality and morbidity in this population. Endothelial dysfunction is crucial for the progression of these diseases and frequently associated with uremic toxin accumulation, inflammation, and vascular calcification^1^.

MicroRNAs (miRNAs) are small non-coding RNAs with regulatory functions in gene and protein expression. The synthesis and release of miRNAs alters cell physiology, as miRNAs can bind to messenger RNA (mRNA), inducing its degradation and preventing expression of molecules^2^. miRNAs participate in intercellular communication and are widely found in biological fluids. Furthermore, miRNAs can be used in personalized medicine to evaluate disease progression, treatment response, and prognosis. These molecules serve as biomarkers due to their stability and wide variety, and the assessment of their expression levels is highly sensitive and non-invasive^3^.

There are several studies regarding the presence of miRNAs in biological fluids related to CKD or CVD^4^. In this sense, understanding the role of these miRNAs in endothelial activation and dysfunction processes may enable the identification of biomarkers for disease monitoring, which in turn would contribute to early interventions, and the prevention of late processes of atherosclerosis and vascular calcification, in addition to the CKD progression^5, 6^.

## miRNAs and Endothelial Dysfunction

miRNAs in endothelial cells target key signaling pathways involved in crucial cell steps, such as cell cycle, inflammation, migration, proliferation, angiogenesis, oxidative stress, apoptosis, and nitric oxide (NO) signaling^7^. In CKD, studies indicate that several miRNAs may be related to endothelial dysfunction and damage, being part of processes that are strongly linked to cardiovascular events.

Kumar et al.^8^ demonstrated that decreased miR-145 and miR-155 levels are correlated with endothelial dysfunction and CKD severity, which may suggest an increased risk for the development of CVD. Both miRNAs were significantly lower in CKD patients compared with healthy controls. miR-145 is the most prevalent miRNA in vascular walls and involved in regulating vascular smooth muscle cell development^9, 10^. miR-155 is associated with abnormal angiotensin signaling and estimated glomerular filtration rate (eGFR)^11^. Specifically, miR-155 targets the AT1 receptor, preventing its expression. Thus, decreased miR-155 levels in patients with CKD activate the AT1 receptor, a crucial step in the development of renal fibrosis and CVD^12^.

miR-126 is produced by endothelial cells and has atheroprotective and angiogenic activity. Harris et al.^13^ demonstrated that miR-126 suppresses expression of vascular cell adhesion molecule 1 (VCAM-1), an important molecule involved in leukocyte adhesion and inflammation. Since healthy endothelium produces miR-126, the function of this miRNA is thought to be related to the suppression of inflammation in these cells, as well as vascular homeostasis. Wang et al.^14^ showed that circulating miR-126 levels are significantly lower in patients with end-stage CKD compared with healthy controls. miR-126 acts as a modulator when associated with aging and cellular senescence, regulating hypoxia-inducible factor 1 (HIF-1), an important transcriptional activator responsible for cell adaptation to hypoxic stress^15^.

Schober et al.^16^ demonstrated that miR-126-5p promotes endothelial proliferation and limits atherosclerosis through suppression of Notch1 inhibitor delta-like 1 homolog (Dlk1) in mice. Endothelial-to-mesenchymal transition (EndMT) is an important fibrotic process where cells stop expressing their endothelial markers and start expressing mesenchymal markers. This transition can be induced by proteins from the growth factor family and is closely related to cardiac and renal fibrosis. Jordan et al.^17^ showed that there was a decrease in miR-126-3p expression in cells undergoing EndMT. Furthermore, overexpression of this miRNA was able to prevent EndMT by maintaining endothelial markers and inhibiting fibronectin expression. miR-126-3p expression is restricted to endothelial cells and the analysis of its levels may be useful for evaluating endothelium health. In addition, Ohta et al.^18^ demonstrated that interleukin-6 (IL-6) was able to promote endothelial cell adhesion through miR-126-3p suppression, demonstrating the protective aspect that this miRNA has on the endothelium.

Inflammation is an important contributor to endothelial dysfunction and damage, and several studies have demonstrated the role and function of miRNAs in inflammation. For example, Yang et al.^19^ showed that miR-216a promotes M1 macrophage polarization, which leads to atherosclerosis progression. Macrophages have phenotypic heterogeneity and plasticity in inflammatory processes and atherosclerotic plaques. They can be generally divided into two subtypes: an inflammatory subtype (M1) and an anti-inflammatory subtype (M2). According to Yang et al.^19^, miR-215a promotes polarization towards the inflammatory subtype, reducing macrophage anti-inflammatory polarization. Moreover, the study found that plasma mir-216a levels were elevated in patients with vulnerable coronary plaques.

**Figure 1.**
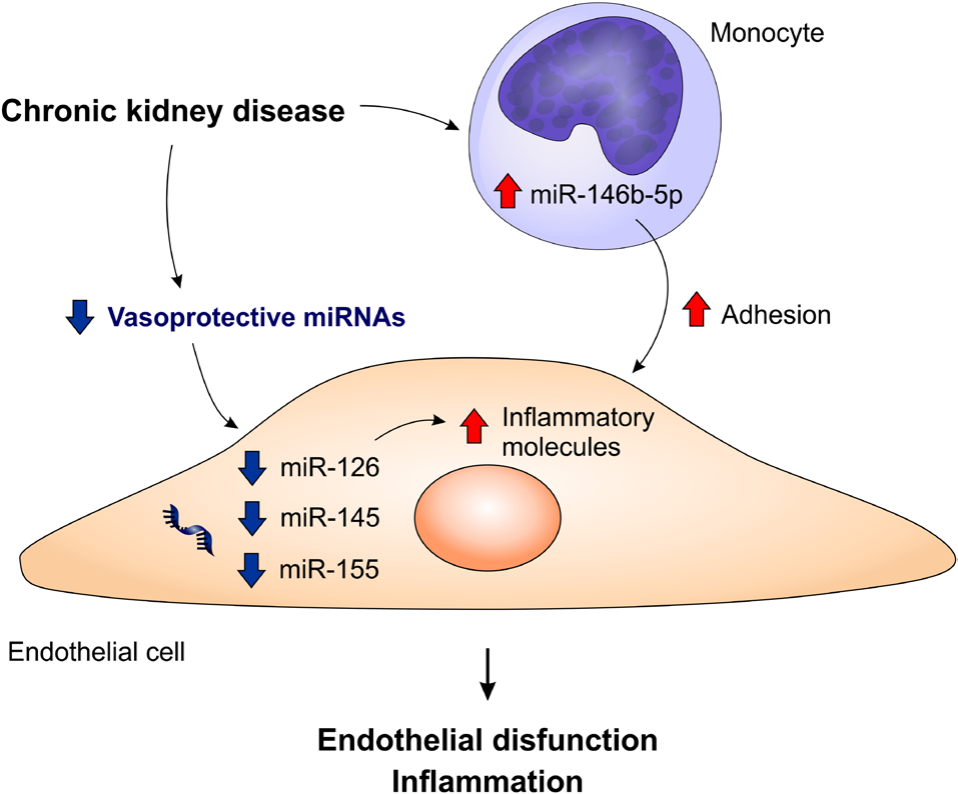
microRNAs involved in endothelial dysfunction and vascular inflammation. The miRNAs with a vasoprotective function (miR-126, miR-145, and miR-155) are reduced in CKD. The decrease in miR-126 levels is associated with an increase in the levels of pro-inflammatory molecules. The uremic environment also contributes to elevated levels of miR-146b-5p in monocytes, increasing their adhesion to the endothelium.

Carmona et al.^20^ showed an increase in miR-145b-5p in the THP-1 monocyte cell line upon treatment with indoxyl sulfate and *p*-cresol, two uremic toxins commonly increased in patients with CKD. Studies demonstrate that mi-146b-5p is related to cellular senescence, and senescent monocytes have been describe to have an increased ability to interact with endothelial cells^21, 22^. Thus, monocyte adhesion to the endothelium may increase, favoring the formation of atherosclerotic plaques in patients with CKD. A summary of miRNAs related to endothelial dysfunction and vascular inflammation is shown in Figure 1.

Oxidative stress also plays an important role in endothelial dysfunction and damage. Liu et al.^23^ in an *in vitro* study demonstrated that inhibition of miR-92a expression and consequent regulation of the Nrf2-KEAP1-ARE signaling pathway can suppress oxidative stress and inflammation, which leads to inhibition of endothelial cell apoptosis and increased cell proliferation. Liu et al.^24^ showed that miR-181a is overexpressed in atherosclerotic plaques and involved in endothelial dysfunction through oxidative stress by regulating Bcl-2, an important anti-apoptotic protein.

## miRNAs Involved in Vascular Calcification

One of the main complications in CKD progression is vascular calcification, a process characterized by calcium deposition in blood vessel walls. Calcification causes arteries to lose elasticity and is a risk factor for CVDs. Studies over the last decade have shown that miRNAs play a key effector role in the pathogenesis of vascular calcification, especially in relation to the osteogenic differentiation of vascular smooth muscle cells.

The differentiation of vascular smooth muscle cells into an osteoblastic phenotype is characterized by Runx2 transcription factor activation, inducing the expression of several calcification-related genes, including osteocalcin (OCN), a key promoter of extracellular matrix mineralization. miR-133a can suppress the Runx2 pathway, thereby inhibiting vascular calcification. Li et al.^25^ found reduced miR-133a levels in histological sections of arteries with calcification from patients with advanced CKD compared to arteries from the control group (without kidney disease). The Runx2 pathway is also negatively regulated by miR-204-5p. Lin et al.^26^ demonstrated that hyperphosphatemia increases Runx2 expression and vascular calcification markers, while simultaneously reducing miR-204-5p levels in vascular smooth muscle cells. However, vascular calcification markers are reduced with increased miR-204-5p expression, even in hyperphosphatemia conditions. Additionally, the authors observed lower miR-204-5p levels in arteries from animals subjected to 5/6 nephrectomy and in arteries from patients with advanced CKD (N = 10) compared to the control group (without kidney damage)^26^.

miR-223-3p is another miRNA with an inhibitory role in vascular calcification. Han et al.^27^ reported that mice with 5/6 nephrectomy and a high phosphate diet had increased miR-223-3p levels in arteries compared to the control group (without kidney disease). Despite this increase, the authors observed that miR-223-3p-deficient animals had worsening medial and atherosclerotic calcification. Furthermore, patients with CKD stages 4 and 5 (N = 45) had lower miR-223-3p levels compared to healthy individuals (N = 34)^28^.

The role of miR-155-5p in vascular calcification under uremic conditions remains unclear, with varying results reported to date. In an *in vitro* study conducted by He et al.^29^, it was shown that the uremic toxin indoxyl sulfate induced miR-155-5p expression in vascular smooth muscle cells. The authors observed that this miRNA negatively regulates matrix Gla protein (MGP) expression, which plays a crucial role in inhibiting the mineralization process. Histological analysis also showed a decrease in MGP levels in arteries with calcification in patients with CKD^29^. These data suggest that indoxyl sulfate promotes vascular calcification by, at least in part, activating the miR-155-5p pathway. On the other hand, Zhao et al.^30^ reported a decrease in miR-155-5p levels in vascular smooth muscle cells exposed to high inorganic phosphate concentrations. Interestingly, the osteogenic differentiation of these cells induced by hyperphosphatemia was attenuated with increased miR-155-5p levels. Table 1 summarizes the main changes in miRNA levels in the uremic environment.

**Table 1:** microRNAs and vascular dysfunction in chronic kidney disease

miRNA	Study	Main findings	Reference
Endothelial dysfunction			
miR-145	Patients with CKD (N = 60)	Reduced serum miR-145 levels in patients with CKD	Kumar et al. (2024)^7^
miR-155	Patients with CKD (N = 60)	Reduced serum miR-155 levels in patients with CKD	Kumar et al. (2024)^7^
	Vascular fibroblasts	miR-155 regulates the angiotensin 1 (AT1) receptor expression	Zheng et al. (2010)^10^
miR-126	Endothelial cells	miR-126 inhibits the expression of vascular adhesion molecule 1 (VCAM-1)	Harris et al. (2008)^12^
	Patients with end-stage CKD (N = 30)	Reduced serum miR-126 levels in patients with end-stage CKD	Wang et al. (2012)^13^
miR-2016a	THP-1 monocytes	miR-216a promotes M1 polarization of macrophages, leading to increased inflammation and the risk of atherosclerosis	Yang et al. (2019)^18^
	Patients with coronary heart disease (N = 518)	High serum miR-2016a levels in patients with vulnerable coronary plaques	Yang et al. (2019)^18^
miR-146b-5p	THP-1 monocytes	Increased miR-146b-5p levels after exposure to indoxyl sulfate and p-cresol uremic toxins.	Carmona et al. (2023)^19^
miR-92a	Endothelial cells	Inhibition of miR-92a may suppress oxidative stress and inflammation in endothelial cells	Liu et al. (2017)^22^
Vascular calcification			
miR-133a	Patients with CKD (N = 6)	Reduced miR-133a levels in calcified arteries of patients with CKD verified by analysis of histological sections	Li et al. (2021) ^24^
miR-204-5p	Vascular smooth muscle cells	Reduced miR-204-5p levels in cells exposed to hyperphosphatemia	Lin et al. (2018) ^25^
	Animals with 5/6 nephrectomy	Reduced miR-204-5p levels in the arteries of animals with 5/6 nephrectomy	Lin et al. (2018) ^25^
	Patients with CKD (N = 10)	Reduced miR-204-5p levels in the arteries of patients with CKD	Lin et al. (2018) ^25^
miR-223-3p	Patients with CKD (N = 45)	Reduced serum miR-223-3p levels in patients with CKD	Ulbing et al. (2017) ^27^
miR-155-5p	Vascular smooth muscle cells	Increased miR-155-5p levels in cells exposed to indoxyl sulfate	He et al. (2020) ^28^
	Vascular smooth muscle cells	Reduced miR-155-5p levels in cells exposed to hyperphosphatemia	Zhao et al. (2021) ^29^

## miRNAS Derived from Extracellular Vesicles

miRNAs are transported from one cell to another via extracellular vesicles, which are nanostructures that have a lipid membrane and are released by cells under physiological and pathological conditions. The content of these vesicles depends on the cell of origin and pathophysiological conditions to which they have been exposed. Once released into the extracellular environment, extracellular vesicles can be taken up by other cells, playing an important role in intercellular communication.

Koide et al.^31^ evaluated the miRNA content of circulating extracellular vesicles isolated from the blood of patients with CKD (N = 37) using transcriptomic analysis. These vesicles showed decreased miR-16-5p, miR-17-5p, miR-20a-5p, and miR-106b-5p levels, especially in more advanced stages of the disease. Reduced levels of these four miRNAs were also observed in animals with adenine-induced tubulointerstitial fibrosis compared to animals without kidney damage. Koide et al.^31^ reported that these four miRNAs, especially miR-16-5p, suppress VEGFA, which is related to osteogenic gene activation in vascular smooth muscle cells. These data suggest that low expression of these miRNAs contributes to vascular calcification by failing to suppress the VEGFA pathway. In addition, the authors reported that reduced miR-16-5p, miR-17-5p, miR-20a-5p, and miR-106b-5p levels in circulating extracellular vesicles is a predictive indicator of aortic vascular calcification in patients with CKD.

Regarding the vascular microenvironment, extracellular vesicles derived from endothelial cells or other cells within the vessel may have a paracrine effect on vascular smooth muscle cells. Lin et al.^32^ demonstrated that extracellular vesicles from endothelial cells exposed to hyperphosphatemia showed high miR-670-3p levels, a miRNA related to vascular calcification. Thus, extracellular vesicles were enriched with miR-670-3p and applied intravenously to animals with 5/6 nephrectomy, which caused an increase in arterial calcification. Increased miR-670-3p levels were also observed in extracellular vesicles isolated from the plasma of stage 5 CKD patients (N = 15) compared to healthy individuals^32^. In another study evaluating the vascular microenvironment, Zheng et al.^33^ observed that hyperphosphatemia induces production of extracellular vesicles by adventitial fibroblasts with high miR-21-5p levels, which promotes osteogenic differentiation of smooth muscle cells and consequently vascular calcification.

**Figure 2.**
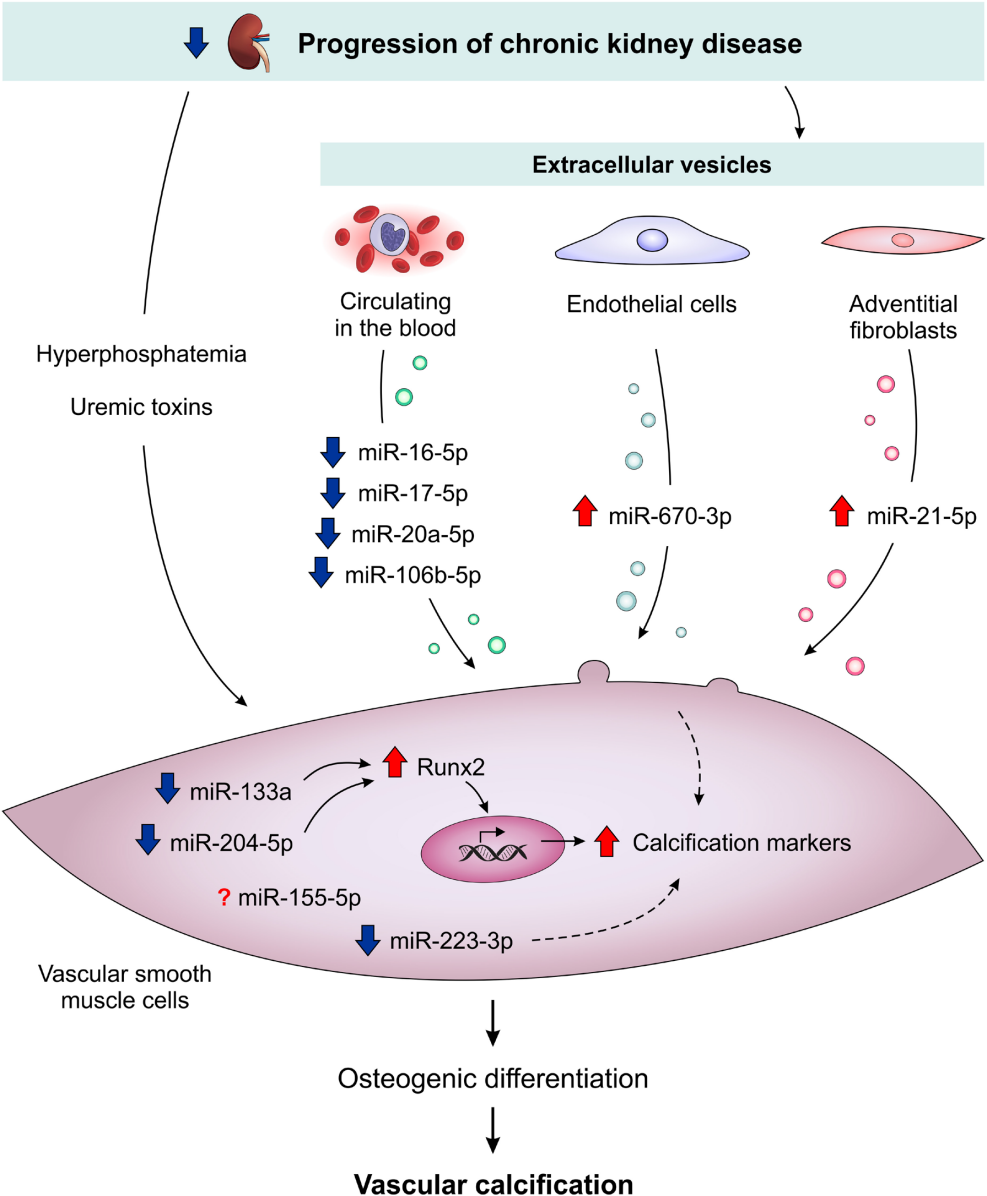
microRNAs that contribute to the osteogenic differentiation of vascular smooth muscle cells in CKD. The uremic environment promotes a reduction in miR-133a and miR-204-5p levels, which is related to an increase in Runx2 and calcification markers. Reduced miR-223-3p levels are also observed in CKD, and it is suggested that this decrease contributes to the calcification process. The miRNA content of extracellular vesicles from blood, endothelial cells and fibroblasts is altered in uremic conditions, inducing vascular smooth muscle differentiation.

Figure 2 shows the main miRNAs involved in osteogenic differentiation in vascular smooth muscle cells in CKD.

## miRNAs and Diabetic Kidney Disease

Diabetic kidney disease (DKD) is one of the major complications of diabetes and a leading cause of end-stage renal disease. DKD is characterized by excessive synthesis of extracellular matrix proteins, which are deposited in the glomeruli and tubular interstitium, leading to glomerular necrosis and interstitial fibrosis^34^. Some studies have shown that various miRNAs may be involved in renal fibrosis, acting as either anti-fibrotic or pro-fibrotic factors.

The let-7 miRNA family has been described as negative regulators of renal fibrosis. Renal levels of let-7 were decreased in the unilateral urethral obstruction (UUO) model in mice, where a positive regulation of TGF-β is usually found. The let-7 family decreases the expression of extracellular matrix proteins through mechanisms that involve signaling by TGF-β^35, 36^. let-7 family has also been described to have effects on endothelial-to-mesenchymal transition (EndMT), an important mechanism by which endothelial cells lose their endothelial phenotype and acquire mesenchymal characteristics, becoming more invasive and fibroblast-like^37^.

miR-29 has also been shown to have antifibrotic activity in kidneys^38, 39^. Du et al.^40^ demonstrated that miR-29a levels were reduced by high glucose or TGF-β in human proximal tubule cells (HK-2), and this reduction was associated with an increase in collagen IV production. In animal studies, it was shown that miR-29 expression decreased as renal fibrosis progressed in mice with unilateral ureteral obstruction. The overexpression of miR-29b inhibited induction of TGF-β-mediated collagen I and III in tubular epithelial cells, while the knockdown of this miRNA increased the expression of these genes^41^.

miR-21 has been extensively studied due to its relevant targets for dopa-responsive dystonia and fibrosis, especially related to TGF-β^42, 43^. Studies have shown that TGF-β leads to an up-regulation of miR-21 in the liver, heart and kidneys in rats, being associated with TGF-β-induced fibrosis in these organs^43, 44, 45, 46^. McClelland et al.^47^ demonstrated that up-regulation of miR-21 in the kidneys was positively associated with the severity of fibrosis and renal dysfunction in patients with DKD.

## Potential use of miRNAs in Clinical Practice

Since their discovery, miRNAs have been studied in a wide range of diseases, including CKD, where they play a crucial role in regulating pathologic processes like inflammation, fibrosis, and vascular damage. miRNAs offer promising clinical perspectives, both as biomarkers and potential therapeutical targets. The stability of miRNA in biological fluids, such as serum, urine, and saliva, make them good biomarkers as these samples are easily obtained samples and analyze.

The profiling of circulating miRNAs is a promising scenario in many diseases, including CKD. For example, miR-223 was identified as a potential biomarker for CKD progression, as its plasma levels directly correlated with eTGF, systemic inflammation, and vascular damage^48, 49^. Detection of urinary miRNAs, such as miR-29 and miR-30, has been shown to correlate with acute and chronic kidney damage and can serve as a predictive tool for disease progression^50^. Another relevant example is miR-200c, which plays a role in controlling epithelial-mesenchymal transition and has been found to be high in patients with advanced CKD. Abnormal regulation of miRNAs such as miR-200c may be an early progression marker for fibrosis and kidney injury^51^. Abdel-Tawab et al.^52^ demonstrated that patients with DKD have significantly high serum levels of miR-221. Diabetic patients without kidney disease also showed higher levels compared to patients with kidney disease but no diabetes and healthy individuals. A positive correlation was observed between serum hsa-miR-221 levels and fasting insulin and glucose in DKD patients. The study concluded that miR-221 levels have potential to be valuable biomarker for the prognosis and diagnosis of DKD. The clinical use of these biomarkers could enable more accurate stratification of patients, monitoring disease progression, and adjusting of therapies according to individual risk profiles.

Cai et al.^10^ demonstrated that microRNA-145 is involved in the pathogenesis of renal vascular lesions in patients with juvenile lupus nephritis, with altered levels of this miRNA correlating with the severity of the lesions, suggesting that miR-145 modulation may be a promising therapeutic strategy to improve the renal and vascular health of these patients. The use of nanoparticles and other innovative delivery systems has recently been investigated to improve the efficiency of miRNA-targeting tissues^53^. Anti-miRNAs, synthetic oligonucleotides that inhibit the function of specific miRNAs, are at an advanced stage of research for therapeutic use. Recent clinical trials targeting miR-21 using anti-miRNAs have shown a significant reduction in the progression of renal fibrosis with improvement in renal function^54^. These results highlight the clinical potential of miRNAs as therapeutic targets, especially in the context of CKD, where therapeutic options are limited. Another recent advance is the use of miRNA mimetics, which can restore normal regulatory miRNA levels that are decreased in diseases such as CKD. For example, miR-126 mimetics have been successfully tested in animal models of CKD-associated cardiovascular disease, resulting in improved endothelial function^55^.

Despite the great potential of miRNAs in managing CKD and its vascular complications, several limitations need to be overcome before their widespread clinical implementation. One of the main difficulties lies in the specificity and selectivity of miRNA delivery to target cells. Although innovative methods, such as nanoparticles and viral vectors, are being developed, there are risks associated with nonspecific delivery, which can result in off-target effects and the modulation of untargeted genes, causing systemic adverse effects^53^. Furthermore, the complex regulation of miRNAs, which can act on multiple biological pathways simultaneously, increases the possibility of unexpected side effects. Another important obstacle is the heterogeneity of patients, since factors such as age, comorbidities, and kidney disease stage can influence miRNA expression and efficacy, limiting the use of a single therapeutic approach for all patients.

There are also challenges in using miRNAs as biomarkers. Although they are stable in biological fluids, standardization of detection methods is still a problem. Variations in miRNA collection, storage, and analysis techniques can impact the accuracy and reproducibility of results, making their clinical validation difficult. In addition, identifying the specific miRNA profiles in different CKD stages and its cardiovascular complications still requires further investigation to avoid misdiagnoses^56^.

Multicenter studies with diverse populations and large patient samples are needed to advance in the field of miRNAs and effectively translate them into clinical practice. Studies of this magnitude are essential to validate preclinical findings and initial trials in a real-world setting, ensuring that the results apply to different populations and at different CKD stages.

## Conclusion

MiRNAs play a key role in the cellular mechanisms involved in endothelial dysfunction and vascular calcification, processes that are intrinsically related to CKD progression and development of CVDs. Analysis of the levels of circulating miRNAs, whether associated with extracellular vesicles or not, may be important for evaluating endothelial dysfunction and vascular damage in patients with CKD. These studies may provide valuable data, both as biomarkers and as potential therapeutic targets, aiming to prevent cardiovascular complications. The use of miRNAs in the clinic, especially for the management of CKD and its vascular complications, has significant potential. miRNAs offer a promising tool as biomarkers for early diagnosis and prognosis, allowing more personalized and effective interventions. Advances in development of miRNA-based therapies suggest that these small gene regulators may play a crucial role in the treatment of CKD and its complications in the future. However, further clinical studies are needed to ensure the efficacy and safety of these approaches, especially regarding the selective delivery of miRNA-based therapies without adverse effects.

## Data Availability

The dataset supporting the findings of this study is not publicly available.

## References

[B1] Stevens PE, Ahmed SB, Carrero JJ, Foster B, Francis A, Hall RK (2024). KDIGO 2024 Clinical Practice Guideline for the Evaluation and Management of Chronic Kidney Disease. Kidney Int.

[B2] Jorge AL, Pereira ER, Oliveira CS (2021). MicroRNAs: understanding their role in gene expression and cancer. Einstein (Sao Paulo).

[B3] Stopic B, Dragicevic S, Medic-Brkic B, Nikolic A, Stojanovic M, Budisavljevic S (2021). Biomarkers of Uremic Cardiotoxicity. Toxins (Basel).

[B4] Motshwari DD, Matshazi DM, Erasmus RT, Kengne AP, Matsha TE, George C (2023). MicroRNAs associated with chronic kidney disease in the general population and high-risk subgroups: a systematic review. Int J Mol Sci.

[B5] Garmaa G, Bunduc S, Kói T, Hegyi P, Csupor D, Ganbat D (2024). A systematic review and meta-analysis of microrna profiling studies in chronic kidney diseases. Noncoding RNA.

[B6] Zou C. (2023). Advances in the study of miRNAs in chronic kidney disease with cardiovascular complications. Front Physiol.

[B7] Piao X, Ma L, Xu Q, Zhang X, Jin C (2023). Noncoding RNAs: versatile regulators of endothelial dysfunction. Life Sci.

[B8] Kumar A, Priyadarshini G, Parameswaran S, Ramesh A, Rajappa M (2024). Evaluation of MicroRNA 145 and MicroRNA 155 as markers of cardiovascular risk in chronic kidney disease. Cureus.

[B9] Taïbi F, Metzinger-Le Meuth V, M’Baya-Moutoula E, Djelouat M, Louvet L, Bugnicourt JM (2014). Possible involvement of microRNAs in vascular damage in experimental chronic kidney disease. Biochim Biophys Acta.

[B10] Cai Z, Xiang W, Peng X, Ding Y, Liao W, He X (2019). MicroRNA-145 involves in the pathogenesis of renal vascular lesions and may become a potential therapeutic target in patients with juvenile lupus nephritis. Kidney Blood Press Res.

[B11] Zheng L, Xu CC, Chen WD, Shen WL, Ruan CC, Zhu LM (2010). MicroRNA-155 regulates angiotensin II type 1 receptor expression and phenotypic differentiation in vascular adventitial fibroblasts. Biochem Biophys Res Commun.

[B12] Chen NX, Kiattisunthorn K, O’Neill KD, Chen X, Moorthi RN, Gattone VH (2013). Decreased MicroRNA is involved in the vascular remodeling abnormalities in Chronic Kidney Disease (CKD). PLoS One.

[B13] Harris TA, Yamakuchi M, Ferlito M, Mendell JT, Lowenstein CJ (2008). MicroRNA-126 regulates endothelial expression of vascular cell adhesion molecule 1. Proc Natl Acad Sci USA.

[B14] Wang H, Peng W, Shen X, Huang Y, Ouyang X, Dai Y (2012). Circulating levels of inflammation-associated miR-155 and endothelial-enriched miR-126 in patients with end-stage renal disease. Braz J Med Biol Res.

[B15] Alique M, Bodega G, Giannarelli C, Carracedo J, Ramírez R (2019). MicroRNA-126 regulates Hypoxia-Inducible Factor-1α which inhibited migration, proliferation, and angiogenesis in replicative endothelial senescence. Sci Rep.

[B16] Schober A, Nazari-Jahantigh M, Wei Y, Bidzhekov K, Gremse F, Grommes J (2014). MicroRNA-126-5p promotes endothelial proliferation and limits atherosclerosis by suppressing Dlk1. Nat Med.

[B17] Jordan NP, Tingle SJ, Shuttleworth VG, Cooke K, Redgrave RE, Singh E (2021). MiR-126-3p is dynamically regulated in endothelial-to-mesenchymal transition during fibrosis. Int J Mol Sci.

[B18] Ohta M, Kihara T, Toriuchi K, Aoki H, Iwaki S, Kakita H (2020). IL-6 promotes cell adhesion in human endothelial cells via microRNA-126-3p suppression. Exp Cell Res.

[B19] Yang S, Li J, Chen Y, Zhang S, Feng C, Hou Z (2019). MicroRNA-216a promotes M1 macrophages polarization and atherosclerosis progression by activating telomerase via the Smad3/NF-κB pathway. Biochimica et Biophysica Acta (BBA) -. Biochim Biophys Acta Mol Basis Dis.

[B20] Carmona A, Guerrero F, Muñoz-Castañeda JR, Jimenez MJ, Rodriguez M, Soriano S (2023). Uremic Toxins Induce THP-1 monocyte endothelial adhesion and migration through specific miRNA expression. Int J Mol Sci.

[B21] Merino A, Buendia P, Martin-Malo A, Aljama P, Ramirez R, Carracedo J (2011). Senescent CD14+CD16+ monocytes exhibit proinflammatory and proatherosclerotic activity. J Immunol.

[B22] Yanaka M, Honma T, Sato K, Shinohara N, Ito J, Tanaka Y (2011). Increased monocytic adhesion by senescence in human umbilical vein endothelial cells. Biosci Biotechnol Biochem.

[B23] Liu H, Wu HY, Wang WY, Zhao ZL, Liu XY, Wang LY (2017). Regulation of miR-92a on vascular endothelial aging via mediating Nrf2-KEAP1-ARE signal pathway. Eur Rev Med Pharmacol Sci.

[B24] Liu G, Li Y, Gao XG (2016). microRNA-181a is upregulated in human atherosclerosis plaques and involves in the oxidative stress-induced endothelial cell dysfunction through direct targeting Bcl-2. Eur Rev Med Pharmacol Sci.

[B25] Li S, Zhi F, Hu M, Xue X, Mo Y (2022). MiR-133a is a potential target for arterial calcification in patients with end-stage renal disease. Int Urol Nephrol.

[B26] Lin X, Xu F, Cui RR, Xiong D, Zhong JY, Zhu T (2018). Arterial calcification is regulated via an miR-204/DNMT3a regulatory circuit both in vitro and in female mice. Endocrinology.

[B27] Han Y, Zhang J, Huang S, Cheng N, Zhang C, Li Y (2021). MicroRNA-223-3p inhibits vascular calcification and the osteogenic switch of vascular smooth muscle cells. J Biol Chem.

[B28] Ulbing M, Kirsch AH, Leber B, Lemesch S, Münzker J, Schweighofer N (2017). MicroRNAs 223-3p and 93-5p in patients with chronic kidney disease before and after renal transplantation. Bone.

[B29] He X, Wang Z, Wei L, Cheng X, Chen L, Gao F (2020). Indoxyl sulfate promotes osteogenic differentiation of vascular smooth muscle cells by miR-155-5p-dependent downregulation of matrix Gla protein via ROS/NF-κB signaling. Exp Cell Res.

[B30] Zhao J, Liu Z, Chang Z. (2021). Osteogenic differentiation and calcification of human aortic smooth muscle cells is induced by the RCN2/STAT3/miR-155-5p feedback loop. Vascul Pharmacol.

[B31] Koide T, Mandai S, Kitaoka R, Matsuki H, Chiga M, Yamamoto K (2023). Circulating extracellular vesicle-propagated microRNA signature as a vascular calcification factor in chronic kidney disease. Circ Res.

[B32] Lin X, Shan SK, Xu F, Zhong JY, Wu F, Duan JY (2022). The crosstalk between endothelial cells and vascular smooth muscle cells aggravates high phosphorus-induced arterial calcification. Cell Death Dis.

[B33] Zheng MH, Shan SK, Lin X, Xu F, Wu F, Guo B (2023). Vascular wall microenvironment: exosomes secreted by adventitial fibroblasts induced vascular calcification. J Nanobiotechnology.

[B34] Sakuma H, Hagiwara S, Kantharidis P, Gohda T, Suzuki Y (2020). Potential targeting of renal fibrosis in diabetic kidney disease using MicroRNAs. Front Pharmacol.

[B35] Brennan EP, Nolan KA, Börgeson E, Gough OS, McEvoy CM, Docherty NG (2013). Lipoxins attenuate renal fibrosis by inducing let-7c and suppressing TGFβR1. J Am Soc Nephrol.

[B36] Wang B, Jha JC, Hagiwara S, McClelland AD, Jandeleit-Dahm K, Thomas MC (2014). Transforming growth factor-β1-mediated renal fibrosis is dependent on the regulation of transforming growth factor receptor 1 expression by let-7b. Kidney Int.

[B37] Chang CJ, Hsu CC, Chang CH, Tsai LL, Chang YC, Lu SW (2011). Let-7d functions as novel regulator of epithelial-mesenchymal transition and chemoresistant property in oral cancer. Oncol Rep.

[B38] van Rooij E, Sutherland LB, Thatcher JE, DiMaio JM, Naseem RH, Marshall WS (2008). Dysregulation of microRNAs after myocardial infarction reveals a role of miR-29 in cardiac fibrosis. Proc Natl Acad Sci USA.

[B39] Xiao J, Meng XM, Huang XR, Chung AC, Feng YL, Hui DS (2012). miR-29 inhibits bleomycin-induced pulmonary fibrosis in mice. Mol Ther.

[B40] Du B, Ma LM, Huang MB, Zhou H, Huang HL, Shao P (2010). High glucose down-regulates miR-29a to increase collagen IV production in HK-2 cells. FEBS Lett.

[B41] Qin W, Chung ACK, Huang XR, Meng XM, Hui DS, Yu CM (2011). TGF-β/Smad3 signaling promotes renal fibrosis by inhibiting miR-29. J Am Soc Nephrol.

[B42] Godwin JG, Ge X, Stephan K, Jurisch A, Tullius SG, Iacomini J (2010). Identification of a microRNA signature of renal ischemia reperfusion injury. Proc Natl Acad Sci USA.

[B43] Zhong X, Chung ACK, Chen HY, Meng XM, Lan HY (2011). Smad3-Mediated Upregulation of miR-21 Promotes Renal Fibrosis. J Am Soc Nephrol.

[B44] Zavadil J, Narasimhan M, Blumenberg M, Schneider RJ (2007). Transforming growth factor-β and microRNA:mRNA regulatory networks in epithelial plasticity. Cells Tissues Organs.

[B45] Davis BN, Hilyard AC, Lagna G, Hata A (2008). SMAD proteins control DROSHA-mediated microRNA maturation. Nature.

[B46] Loboda A, Sobczak M, Jozkowicz A, Dulak J (2016). TGF-β1/Smads and miR-21 in renal fibrosis and inflammation. Mediators Inflamm.

[B47] McClelland AD, Herman-Edelstein M, Komers R, Jha JC, Winbanks CE, Hagiwara S (2015). miR-21 promotes renal fibrosis in diabetic nephropathy by targeting PTEN and SMAD7. Clin Sci (Lond).

[B48] Fourdinier O, Schepers E, Metzinger-Le Meuth V, Glorieux G, Liabeuf S, Verbeke F (2019). Serum levels of miR-126 and miR-223 and outcomes in chronic kidney disease patients. Sci Rep.

[B49] Fujii R, Yamada H, Yamazaki M, Munetsuna E, Ando Y, Ohashi K (2019). Circulating microRNAs (miR-126, miR-197, and miR-223) are associated with chronic kidney disease among elderly survivors of the Great East Japan Earthquake. BMC Nephrol.

[B50] Mukhadi S, Hull R, Mbita Z, Dlamini Z (2015). The role of MicroRNAs in kidney disease. Noncoding RNA.

[B51] Singh BMK, Mathew M (2022). Epithelial-mesenchymal transition and its role in renal fibrogenesis. Braz Arch Biol Technol.

[B52] Abdel-Tawab MS, Mohamed MG, Doudar NA, Rateb EE, Reyad HR, Elazeem NAA (2023). Circulating hsa-miR-221 as a possible diagnostic and prognostic biomarker of diabetic nephropathy. Mol Biol Rep.

[B53] Byun MJ, Lim J, Kim SN, Park DH, Kim TH, Park W (2022). Advances in nanoparticles for effective delivery of RNA therapeutics. Biochip J.

[B54] Gomez IG, MacKenna DA, Johnson BG, Kaimal V, Roach AM, Ren S (2015). Anti-microRNA-21 oligonucleotides prevent Alport nephropathy progression by stimulating metabolic pathways. J Clin Invest.

[B55] Climent M, Quintavalle M, Miragoli M, Chen J, Condorelli G, Elia L (2015). TGFβ triggers miR-143/145 transfer from smooth muscle cells to endothelial cells, thereby modulating vessel stabilization. Circ Res.

[B56] Chen X, Ba Y, Ma L, Cai X, Yin Y, Wang K (2008). Characterization of microRNAs in serum: a novel class of biomarkers for diagnosis of cancer and other diseases. Cell Res.

