# Supplementary material for: MicroRNAs and vascular damage in chronic kidney disease: advances and clinical implications
Source: J Bras Nefrol. 2025 May 19;47(3):e20240223. doi: 10.1590/2175-8239-JBN-2024-0223en (PMC12088645; doi:10.1590/2175-8239-JBN-2024-0223en)
Supplement: Translation

# Translation: MicroRNAs e dano vascular na doença renal crônica: avanços e implicações clínicas

**DOI:** 10.1590/2175-8239-JBN-2024-0223pt

**Authors:** Regiane Stafim da Cunha, Carolina Amaral Bueno Azevedo, Guilherme Miniskiskosky, Paulo Cézar Gregório, Andréa Emilia Marques Stinghen

**Affiliations:** 1 Universidade Federal do Paraná, Laboratório de Nefrologia Experimental, Departamento de Patologia Básica, Curitiba, PR, Brasil.

**Keywords:** Insuficiência Renal Crônica, Disfunção Endotelial, Calcificação Vascular, MicroRNAs

## Abstract

A doença renal crônica (DRC) está intimamente associada à disfunção endotelial e à calcificação vascular, principais contribuintes para o desenvolvimento de doenças cardiovasculares nessa população. Os microRNAs (miRNAs) constituem um grupo de RNAs que não codificam proteínas, mas que atuam na regulação da expressão gênica e de outros processos celulares. Estudos recentes têm demonstrado alterações nos níveis de diversos miRNAs associados à progressão da disfunção renal. Pacientes com DRC apresentam níveis reduzidos de miR-126, um microRNA produzido pelo endotélio e que possui função ateroprotetora. Níveis reduzidos de miRNAs inibidores da calcificação vascular, como o miR-133a e o miR-204-5p, também são encontrados em pacientes com DRC. Essas alterações podem contribuir para o quadro de disfunção vascular nesses pacientes. Portanto, compreender o perfil de microRNAs no contexto da DRC pode ser importante para a identificação de novos biomarcadores e potenciais alvos terapêuticos. Diante da crescente relevância da análise de microRNAs, esta revisão aborda os recentes avanços no estudo de microRNAs relacionados à disfunção vascular na DRC e suas potenciais aplicações na clínica translacional.

## Introdução

A doença renal crônica (DRC) se manifesta pela perda da função renal de forma gradativa e irreversível, tendo como doenças associadas a hipertensão e o diabetes. Complicações e doenças cardiovasculares (DCV) estão intimamente relacionadas à DRC, constituindo a principal causa de mortalidade e morbidade nessa população. Nesse contexto, a disfunção endotelial é proeminente na progressão dessas doenças, associada ao acúmulo de toxinas urêmicas, aos processos inflamatórios decorrentes e à calcificação vascular^1^.

Os microRNAs (miRNAs) são pequenos RNAs que não codificam proteínas, mas que exercem funções regulatórias na expressão gênica e proteica. A síntese e a liberação de miRNAs alteram a fisiologia dos processos celulares. O miRNA pode se ligar a um RNA mensageiro (mRNA) alvo, induzindo sua degradação e impedindo a produção de diferentes moléculas na célula^2^. Os miRNAs servem como veículo de comunicação intercelular e são amplamente encontrados em fluídos biológicos na forma circulante. Eles podem ser utilizados para avaliar o estadiamento, a resposta à terapia e o prognóstico dos pacientes, dentro de uma visão de medicina personalizada. Essas moléculas servem como biomarcadores devido à sua estabilidade e ampla variedade, além da avaliação de seus níveis de expressão ser de alta sensibilidade e não invasiva^3^.

Diversos são os achados relativos à presença de miRNAs em fluidos biológicos e relacionados tanto à DRC quanto às DCV^4^. Nesse sentido, o entendimento do papel desses miRNAs nos processos de ativação e disfunção endotelial pode permitir a identificação de biomarcadores úteis para o monitoramento da doença, contribuindo para intervenções precoces e evitando processos tardios de aterosclerose e de calcificação vascular, além da progressão da DRC^5, 6^.

## miRNAs Relacionados à Disfunção Endotelial

Em células endoteliais, os miRNAS têm como alvo vias de sinalização-chave envolvidas no ciclo celular, inflamação, apoptose, migração, proliferação, angiogênese, estresse oxidativo e sinalização de óxido nítrico (NO)^7^. Na DRC, estudos indicam que diversos miRNAs podem estar relacionados à disfunção e ao dano endotelial, processos fortemente ligados a eventos cardiovasculares.

Kumar et al.^8^ demostraram que níveis reduzidos de miR-145 e miR-155 estão correlacionados com a disfunção endotelial e com a severidade da DRC, o que pode sugerir um aumento no risco para o desenvolvimento de DCV. Ambos os miRNAs estavam consideravelmente diminuídos em pacientes com DRC, quando comparados a controles saudáveis. O miR-145 está envolvido na regulação do desenvolvimento de células musculares lisas vasculares e é o miRNA mais prevalente nas paredes vasculares^9, 10^. Já o miR-155 é associado a anomalias na sinalização da angiotensina e na taxa de filtração glomerular estimada (TFGe)^11^. Mais especificamente, o miR-155 tem como alvo o receptor AT1, prevenindo sua expressão. Dessa forma, em pacientes com DRC, níveis reduzidos de miR-155 ativam o receptor AT1, componente crucial no desenvolvimento de fibrose renal e DCV^12^.

O miR-126 é produzido por células endoteliais e possui atividade ateroprotetora e angiogênica. Harris et al. (2008) demostraram que o miR-126 suprime a expressão da molécula de adesão celular vascular 1 (VCAM-1), uma importante molécula envolvida na adesão de leucócitos e na inflamação. Como o endotélio saudável produz miR-126, sugere-se que a função desse miRNA esteja relacionada à supressão da inflamação nessas células e à homeostase vascular^13^. Wang et al.^14^ demonstraram que os níveis circulantes de miR-126 estão significantemente reduzidos em pacientes com DRC em estágio terminal, quando comparados com controles saudáveis. Quando relacionado com o envelhecimento e a senescência celular, o miR-126 atua como modulador, regulando o fator induzível por hipóxia 1 (HIF-1), um importante ativador transcricional responsável pela adaptação das células ao estresse por hipóxia^15^.

Schober et al.^16^ demonstraram que o miR-126-5p promove a proliferação endotelial e limita a aterosclerose por meio da supressão do homólogo delta-like 1 do inibidor Notch1 (Dlk1) em ratos. Em processos fibróticos, a transição endotélio-mesenquimal (EndMT) é um importante processo no qual as células deixam de expressar seus marcadores endoteliais e passam a expressar marcadores mesenquimais. Essa transição pode ser induzida por proteínas da família dos fatores de crescimento e está intimamente relacionada à fibrose cardíaca e renal. Jordan et al.^17^ demonstraram que, em células submetidas ao processo de EndMT, houve uma diminuição na expressão do miR-126-3p. Por outro lado, a superexpressão desse mesmo miRNA foi capaz de prevenir a EndMT por meio da manutenção dos marcadores endoteliais e reprimindo expressão de fibronectina. A expressão do miR-126-3p é restrita às células endoteliais, e a análise de seus níveis pode ser útil na avaliação do endotélio. Além disso, Ohta et al.^18^ demonstraram que a interleucina-6 (IL-6) foi capaz de promover a adesão em células endoteliais por meio da supressão do miR-126-3p, demonstrando o aspecto protetor desse miRNA sobre o endotélio.

A inflamação é um importante fator contribuinte na disfunção e no dano endotelial e diversos estudos têm demonstrado o papel dos miRNAs nesse cenário. Yang et al.^19^ reportaram que o miR-216a promove uma polarização M1 para macrófagos, o que leva à progressão da aterosclerose. Macrófagos possuem uma heterogeneidade fenotípica e plasticidade em processos inflamatórios e placas ateroscleróticas. De forma geral, podem ser divididos em dois subtipos: um subtipo inflamatório (M1) e um subtipo anti-inflamatório (M2). Dessa forma, de acordo com Yang et al.^19^, o miR-216 promove a polarização para o subtipo inflamatório e reduz sua polarização anti-inflamatória. Além disso, o grupo descreve que os níveis plasmáticos de miR-216a se mostraram elevados em pacientes com placas coronárias vulneráveis.

Carmona et al.^20^ demonstraram um aumento de miR-146b-5p na linhagem de monócitos THP-1 quando estimulados com indoxil sulfato e *p*-cresol, duas toxinas urêmicas comumente encontradas em níveis elevados em pacientes com DRC. Estudos demonstram que o mi-146b-5p está relacionado à senescência celular, sendo descrito que monócitos senescentes apresentam aumento na sua capacidade de interação com células endoteliais^21, 22^. Dessa forma, pode ocorrer um aumento na adesão de monócitos ao endotélio, favorecendo a formação de placas ateroscleróticas em pacientes com DRC. Um esquema sobre os miRNAs relacionados à disfunção endotelial e à inflamação vascular é apresentado na Figura 1.

**Figura 1:** MicroRNAs envolvidos na disfunção endotelial e na inflamação vascular. Os miRNAs com função vasoprotetora (miR-126, miR-145 e miR-155) estão reduzidos na DRC. A diminuição dos níveis de miR-126, especificamente, está relacionada com o aumento dos níveis de moléculas pró-inflamatórias. O ambiente urêmico também contribui para o aumento de miR-146b-5p em monócitos, o que está relacionado à maior adesão dessas células ao endotélio.

O estresse oxidativo também exerce importante contribuição na disfunção e no dano endotelial. Liu et al.^23^ demonstraram, *in vitro*, que a inibição da expressão do miR-92a e a consequente regulação da via de sinalização Nrf2-KEAP1-ARE é capaz de suprimir o estresse oxidativo e a inflamação, resultando na inibição da apoptose de células endoteliais e no aumento da proliferação celular. Liu et al.^24^ também demonstraram que o miR-181a está superexpresso em placas ateroescleróticas e está envolvido na disfunção endotelial por meio do estresse oxidativo, por sua regulação do Bcl-2, uma importante proteína anti-apoptótica.

## miRNAs Envolvidos na Calcificação Vascular

Uma das principais complicações da progressão da DRC é a calcificação vascular, um processo caracterizado pela deposição de cálcio nas paredes dos vasos sanguíneos. A calcificação causa a perda de elasticidade das artérias e é considerada um fator de risco para o desenvolvimento de DCVs. Estudos realizados na última década demonstraram que os miRNAs desempenham um papel efetor-chave na patogênese da calcificação vascular, especialmente no que se refere à diferenciação osteogênica das células musculares lisas vasculares.

A diferenciação das células musculares lisas vasculares para um fenótipo osteoblástico é marcada pela ativação do fator de transcrição Runx2 que induz a transcrição de diversos genes relacionados à calcificação, incluindo a osteocalcina (OCN), responsável por promover a mineralização da matriz extracelular. O miR-133a tem a capacidade de suprimir a via do Runx2 e, dessa forma, regular negativamente a calcificação vascular. Nesse sentido, Li et al.^25^ reportaram a detecção de níveis reduzidos de miR-133a em cortes histológicos de artérias com calcificação provenientes de pacientes com DRC avançada, em comparação com artérias do grupo controle (sem doença renal). A via do Runx2 também é regulada negativamente pelo miR-204-5p. Lin et al.^26^ demonstraram que a hiperfosfatemia promove o aumento de Runx2 e de marcadores de calcificação vascular de forma concomitante à redução dos níveis de miR-204-5p em células musculares lisas vasculares. Entretanto, quando se induz o aumento da expressão de miR-204-5p, os marcadores de calcificação vascular são reduzidos, mesmo em condições de hiperfosfatemia. Além disso, os autores verificaram uma redução dos níveis de miR-204-5p em artérias de animais submetidos à nefrectomia 5/6 e em artérias de pacientes com DRC em estágio avançado (N = 10), em comparação ao grupo controle (sem dano renal)^26^.

Outro miRNA com papel inibidor da calcificação vascular é o miR-223-3p. Han et al.^27^ demonstraram que camundongos com nefrectomia 5/6 e uma dieta rica em fosfato apresentaram aumento nos níveis de miR-223-3p nas artérias, em comparação com o grupo controle (sem doença renal). Apesar desse aumento, os autores observaram que animais com deficiência de miR-223-3p tiveram piora do quadro de calcificação medial e aterosclerótica. Ademais, a análise dos níveis séricos de miRNAs indicou que pacientes com DRC nos estágios 4 e 5 (N = 45) apresentam níveis mais baixos de miR-223-3p quando comparados a indivíduos saudáveis (N = 34)^28^.

Já o papel do miR-155-5p na calcificação vascular em condições urêmicas permanece incerto, com resultados divergentes até o momento. Em um estudo *in vitro* conduzido por He et al.^29^, foi demonstrado que a toxina urêmica indoxil sulfato induziu a expressão do miR-155-5p em células musculares lisas vasculares. Os autores observaram que esse miRNA atua regulando negativamente a expressão da proteína Gla da matriz (MGP), responsável por inibir o processo de mineralização. Em análises histológicas, verificou-se também uma redução nos níveis de MGP em artérias com calcificação de pacientes com DRC^29^. Esses dados sugerem que o indoxil sulfato promove a calcificação vascular, ao menos em parte, por meio da ativação da via do miR-155-5p. Por outro lado, Zhao et al.^30^ relataram uma redução nos níveis de miR-155-5p em células musculares lisas vasculares expostas a concentrações elevadas de fosfato inorgânico. Curiosamente, a diferenciação osteogênica dessas células induzidas pela hiperfosfatemia foi atenuada com o aumento dos níveis de miR-155-5p. A Tabela 1 sintetiza as principais alterações nos níveis de miRNAs no ambiente urêmico.

**Tabela 1.** MicroRNAs e a disfunção vascular na doença renal crônica

miRNA	Estudo	Principais achados	Referência
Disfunção endotelial			
miR-145	Pacientes com DRC (N = 60)	Níveis séricos reduzidos de miR-145 em pacientes com DRC	Kumar et al. (2024)^7^
miR-155	Pacientes com DRC (N = 60)	Níveis séricos reduzidos de miR-155 em pacientes com DRC	Kumar et al. (2024)^7^
	Fibroblastos vasculares	miR-155 regula a expressão do receptor de angiotensina 1 (AT1)	Zheng et al. (2010)^10^
miR-126	Células endoteliais	miR-126 inibe a expressão da molécula de adesão vascular 1 (VCAM-1)	Harris et al. (2008)^12^
	Pacientes com DRC em estágio terminal (N = 30)	Níveis séricos reduzidos de miR-126 em pacientes com DRC em estágio terminal	Wang et al. (2012)^13^
miR-2016a	Monócitos THP-1	miR-216a promove polarização M1 para macrófagos, aumentando a inflamação e o risco de aterosclerose	Yang et al. (2019)^18^
	Pacientes com doença coronariana (N = 518)	Níveis séricos elevados de miR-2016a em pacientes com placas coronárias vulneráveis	Yang et al. (2019)^18^
miR-146b-5p	Monócitos THP-1	Níveis aumentados de miR-146b-5p após estimulação com toxinas urêmicas indoxil sulfato e p-cresol.	Carmona et al. (2023)^19^
miR-92a	Células endoteliais	Inibição do miR-92a é capaz de suprimir o estresse oxidativo e a inflamação em células endoteliais de cordão umbilical	Liu et al. (2017)^22^
Calcificação vascular			
miR-133a	Pacientes com DRC (N = 6)	Níveis reduzidos de miR-133a nas artérias com calcificação de pacientes com DRC por meio de análise de cortes histológicos	Li et al. (2021)^24^
miR-204-5p	Células musculares lisas vasculares	Níveis reduzidos de miR-204-5p em células expostas à hiperfosfatemia	Lin et al. (2018)^25^
	Animais com nefrectomia 5/6	Níveis reduzidos de miR-204-5p nas artérias dos animais com nefrectomia 5/6	Lin et al. (2018)^25^
	Pacientes com DRC (N = 10)	Níveis reduzidos de miR-204-5p nas artérias dos pacientes com DRC	Lin et al. (2018)^25^
miR-223-3p	Pacientes com DRC (N = 45)	Níveis séricos reduzidos de miR-223-3p em pacientes com DRC	Ulbing et al. (2017)^27^
miR-155-5p	Células musculares lisas vasculares	Níveis aumentados de miR-155-5p em células expostas ao indoxil sulfato	He et al. (2020)^28^
	Células musculares lisas vasculares	Níveis reduzidos de miR-155-5p em células expostas à hiperfosfatemia	Zhao et al. (2021)^29^

## miRNAs Derivados de Vesículas Extracelulares

Os miRNAs podem ser transportados de uma célula para outra por meio de vesículas extracelulares, que são nanoestruturas delimitadas por uma membrana lipídica e liberadas pelas células tanto em condições fisiológicas quanto patológicas. O conteúdo dessas vesículas, entretanto, reflete a célula de origem e varia de acordo com a condição fisiopatológica a que foram expostas. Uma vez liberadas no meio extracelular, as vesículas extracelulares podem ser captadas por outras células, desempenhando um papel importante na comunicação intercelular.

Koide et al.^31^ avaliaram o conteúdo de miRNAs presentes em vesículas extracelulares circulantes isoladas do sangue de pacientes com DRC (N = 37), por meio de análise transcriptômica. Essas vesículas apresentaram uma redução nos níveis de miR-16-5p, miR-17-5p, miR-20a-5p e miR-106b-5p, especialmente nos estágios mais avançados da doença. A redução dos níveis desses quatro miRNAs também foi observada em animais com fibrose tubulointersticial induzida por adenina, em comparação com animais sem danos renais. Ao investigar o mecanismo celular desses miRNAs, Koide et al.^31^ verificaram que os quatro miRNAs, principalmente o miR-16-5p, realizam a supressão do VEGFA, que está relacionado à ativação de genes osteogênicos em células musculares lisas vasculares. Esses dados sugerem que a baixa expressão desses miRNAs contribui para a calcificação vascular, ao deixar de suprimir a via do VEGFA. Além disso, os autores reportaram que a redução dos níveis de miR-16-5p, miR-17-5p, miR-20a-5p e miR-106b-5p nas vesículas extracelulares circulantes representa um indicador preditivo de calcificação vascular aórtica em pacientes com DRC.

Em relação ao microambiente vascular, as vesículas extracelulares derivadas de células endoteliais ou de outras células que compõem o vaso podem exercer efeito parácrino sobre as células musculares lisas. Lin et al.^32^ demonstraram que vesículas extracelulares provenientes de células endoteliais expostas à condição de hiperfosfatemia apresentavam níveis elevados de miR-670-3p, um miRNA relacionado com a calcificação vascular. Para avaliar o efeito biológico desse tipo de vesícula, os autores enriqueceram vesículas extracelulares com miR-670-3p e as aplicaram por via intravenosa em camundongos com nefrectomia 5/6, observando aumento da calcificação arterial. Níveis aumentados de miR-670-3p em vesículas extracelulares isoladas do plasma de pacientes com DRC em estágio 5 (N = 15) também foram observados, em comparação com indivíduos saudáveis^32^. Em outro estudo que avaliou o microambiente vascular, Zheng et al.^33^ observaram que a hiperfosfatemia induz a produção de vesículas extracelulares por fibroblastos adventiciais contendo níveis elevados de miR-21-5p, o que promove a diferenciação osteogênica das células musculares lisas e, consequentemente, a calcificação vascular.

A Figura 2 ilustra os principais miRNAs envolvidos com a diferenciação osteogênica em células musculares lisas vasculares na DRC.

**Figura 2:** MicroRNAs que contribuem para a diferenciação osteogênica das células musculares lisas vasculares na DRC. O ambiente urêmico promove a redução dos níveis de miR-133a e miR-204-5p, o que está relacionado ao aumento de Runx2 e de marcadores de calcificação. Níveis reduzidos de miR-223-3p também são observados na DRC, e sugere-se que esse fator contribua para o processo de calcificação. Vesículas extracelulares circulantes, de células endoteliais e de fibroblastos, possuem o conteúdo de miRNAs alterado em condições urêmicas e promovem a diferenciação das células musculares lisas vasculares.

## miRNAs e a Doença Renal Diabética

A doença renal diabética (DRD) é uma das maiores complicações do diabetes e uma das principais causas da doença renal em estágio terminal. A DRD é caracterizada por uma síntese excessiva de proteínas da matriz extracelular, que são depositadas nos glomérulos e no interstício tubular, levando à necrose dos glomérulos e à fibrose intersticial^34^. Alguns estudos demonstraram que diversos miRNAs podem estar envolvidos na fibrose renal, atuando como anti- ou mesmo pró-fibróticos.

A família de miRNAs let-7 foi descrita como reguladora negativa da fibrose renal. Os níveis renais de let-7 estavam reduzidos no modelo de obstrução uretral unilateral (OUU) em camundongos, condição na qual há, geralmente, uma regulação positiva do TGF-β. A família let-7 reduz a expressão de proteínas da matriz extracelular por meio de mecanismos que envolvem a sinalização via TGF-β^35, 36^. Além disso, foi descrito que a família let-7 exerce efeitos sobre a transição endotélio-mesenquimal (EndMT), um mecanismo importante pelo qual células endoteliais perdem seu fenótipo endotelial e assumem fenótipos mesenquimais, tornando-se mais invasivas, semelhantes a fibroblastos^37^.

O miR-29 também demonstrou possuir atividade antifibrótica nos rins^38, 39^. Du et al.^40^ observaram que os níveis de miR-29a estavam reduzidos por elevada glucose ou TGF-β em células de túbulo proximal humanas (HK-2), e essa diminuição estava relacionada ao aumento na produção de colágeno IV. Em estudos animais, observou-se que a expressão do miR-29 diminui gradativamente conforme a progressão da fibrose renal em camundongos com OUU. A superexpressão de miR-29b foi capaz de inibir a indução de colágeno I e III mediada por TGF-β em células epiteliais tubulares, enquanto o *knockdown* desse miRNA levou ao aumento da expressão desses genes^41^.

O miR-21 tem sido amplamente estudado devido aos seus alvos relevantes para a DRD e para a fibrose, especialmente os relacionados ao TGF-β^42, 43^. Além disso, foi demonstrado que o TGF-β promove uma regulação positiva do miR-21 no fígado, coração e rins de ratos, estando associado à fibrose induzida por TGF-β nesses órgãos^43, 44, 45, 46^. McClelland et al.^47^ demonstraram que a regulação positiva do miR-21 nos rins esteve positivamente associada à severidade da fibrose e à disfunção renal em pacientes com DRD.

## Potencial Uso Dos miRNAs na Prática Clínica

Desde sua descoberta, os miRNAs têm sido estudados em uma ampla gama de doenças, incluindo a DRC, na qual desempenham um papel crucial na regulação de processos patológicos como inflamação, fibrose e dano vascular. Este campo emergente oferece perspectivas clínicas promissoras, tanto como biomarcadores quanto como potenciais alvos terapêuticos. A estabilidade dos miRNAs em fluidos biológicos, como soro, urina e saliva, torna-os bons biomarcadores, devido à facilidade na obtenção das amostras e na dosagem laboratorial.

O perfil de miRNAs circulantes se tem mostrado promissor ao refletir o estado patológico de várias doenças, incluindo a DRC. Por exemplo, o miR-223 foi identificado como um biomarcador potencial para a progressão da DRC, com seus níveis plasmáticos correlacionando-se diretamente com a TFGe, bem como com o agravamento da inflamação sistêmica e com o dano vascular^48, 49^. A detecção de miRNAs urinários, como o miR-29 e o miR-30, tem demonstrado correlação com lesão renal aguda e crônica, podendo servir como ferramentas preditivas para o agravamento da doença^50^. Outro exemplo relevante é o miR-200c, que desempenha papel no controle da transição epitélio-mesenquimal e foi encontrado em níveis elevados em pacientes com DRC avançada. A regulação inadequada de miRNAs como o miR-200c pode representar um marcador precoce da progressão para fibrose e lesão renal^51^. Abdel-Tawab et al.^52^ demonstraram que pacientes com DRD apresentam níveis elevados e significativos de miR-221 sérico. Pacientes apenas diabéticos apresentaram níveis elevados em comparação a pacientes com doença renal e sem diabetes e a indivíduos saudáveis. Foram observadas correlações positivas entre os níveis séricos de hsa-miR-221 e os valores de insulina e glicose em jejum em pacientes com DRD. O estudo concluiu que os níveis de miR-221 possuem potencial biomarcador promissor para prognóstico e diagnóstico na DRD. O uso clínico desses biomarcadores permitiria uma estratificação mais precisa dos pacientes, possibilitando o monitoramento da progressão da doença e o ajuste das terapias de acordo com os perfis de risco individuais.

Cai et al.^10^ demonstraram que o microRNA-145 está envolvido na patogênese das lesões vasculares renais em pacientes com nefrite lúpica juvenil, com níveis alterados desse miRNA correlacionando-se com a gravidade das lesões, sugerindo que a modulação do miR-145 pode ser uma estratégia terapêutica promissora para melhorar a saúde renal e vascular desses pacientes. Recentemente, a utilização de nanopartículas e outros sistemas inovadores de entrega tem sido investigada para melhorar a eficiência do direcionamento dos miRNAs aos tecidos^53^. Os anti-miRNA, oligonucleotídeos sintéticos explorados que inibem a função de miRNAs específicos, encontram-se em fase avançada de pesquisa para uso terapêutico. Ensaios clínicos recentes que visaram o miR-21, utilizando anti-miRNA, demonstraram uma redução significativa na progressão da fibrose renal, com melhora na função renal^54^. Esses resultados destacam o potencial clínico dos miRNAs como alvos terapêuticos, especialmente no contexto da DRC, em que as opções terapêuticas são limitadas. Outro avanço recente é o uso de miméticos de miRNAs, que podem restaurar os níveis normais de miRNAs reguladores que se encontram diminuídos em doenças como a DRC. Por exemplo, miméticos de miR-126 foram testados com sucesso em modelos animais de doença cardiovascular associada à DRC, resultando em melhora da função endotelial^55^. Contudo, há desafios importantes a serem superados antes que as terapias baseadas em miRNAs possam ser utilizadas na prática clínica.

Apesar do grande potencial dos miRNAs no manejo da DRC e de suas complicações vasculares, existem diversas limitações que precisam ser superadas antes de sua ampla implementação clínica. Uma das principais dificuldades reside na especificidade e na seletividade de entrega de miRNAs às células-alvo. Embora métodos inovadores, como nanopartículas e vetores virais, estejam em desenvolvimento, há riscos associados à entrega inespecífica, que pode resultar em efeitos *off-target*, modulando genes não desejados e causando efeitos adversos sistêmicos^53^. Além disso, a regulação complexa dos miRNAs, que podem atuar em múltiplas vias biológicas simultaneamente, aumenta a possibilidade de efeitos colaterais inesperados. Outro obstáculo importante é a heterogeneidade entre os pacientes, uma vez que fatores como idade, comorbidades e estágio da doença renal podem influenciar a expressão e a eficácia dos miRNAs, limitando o uso de uma abordagem terapêutica única para todos os pacientes.

Há também desafios relacionados ao uso de miRNAs como biomarcadores. Embora sejam estáveis em fluidos biológicos, a padronização dos métodos de detecção ainda representa um problema. Variações nas técnicas de coleta, armazenamento e análise dos miRNAs podem impactar a precisão e a reprodutibilidade dos resultados, dificultando sua validação clínica. Além disso, a identificação de perfis específicos de miRNAs nos diferentes estágios da DRC e de suas complicações cardiovasculares ainda requer mais investigações, a fim de evitar diagnósticos imprecisos^56^.

Para que os avanços no campo dos miRNAs sejam traduzidos para a prática clínica de forma eficaz, são necessários estudos multicêntricos que envolvam populações diversas e grandes amostras de pacientes. Estudos dessa magnitude são essenciais para validar os achados pré-clínicos e ensaios iniciais em contextos reais, garantindo que os resultados sejam aplicáveis a diferentes populações e em diversos estágios da DRC.

## Conclusão

Os miRNAs desempenham papel fundamental nos mecanismos celulares envolvidos na disfunção endotelial e na calcificação vascular, processos intrinsecamente relacionados à progressão da DRC e ao desenvolvimento de DCVs. A análise dos níveis desses miRNAs circulantes, associados ou não a vesículas extracelulares, pode ser importante para a avaliação da disfunção endotelial e do dano vascular em pacientes com DRC. Esses estudos podem fornecer dados valiosos tanto como biomarcadores quanto como potenciais alvos terapêuticos, visando à prevenção de complicações cardiovasculares. O uso de miRNAs na prática clínica, especialmente no manejo da DRC e de suas complicações vasculares, apresenta potencial significativo. Como biomarcadores, os miRNAs oferecem uma ferramenta promissora para o diagnóstico precoce e o prognóstico, permitindo intervenções mais personalizadas e eficazes. Os avanços no desenvolvimento de terapias baseadas em miRNAs sugerem que esses pequenos reguladores gênicos poderão, no futuro, desempenhar um papel crucial no tratamento da DRC e de suas complicações. No entanto, são necessários mais estudos clínicos para garantir a eficácia e a segurança dessas abordagens, especialmente no que diz respeito à entrega de terapias baseadas em miRNAs, de maneira seletiva e sem efeitos adversos.

## Data Availability

O conjunto de dados que dá suporte aos resultados deste estudo não está disponível publicamente.
